# *Schizymenia jonssonii* sp. nov. (Nemastomatales, Rhodophyta): a relict or an introduction into the North Atlantic after the last glacial maximum?

**DOI:** 10.1111/jpy.12957

**Published:** 2020-01-09

**Authors:** Karl Gunnarsson, Stephen Russell, Juliet Brodie

**Affiliations:** ^1^ Marine and Freshwater Research Institute Skúlagata 4 101 Reykjavík Iceland; ^2^ Department of Life Sciences Natural History Museum Cromwell Road London SW7 5BD UK

**Keywords:** COI, Iceland, molecular phylogeny, phenology, North Atlantic, *rbc*L, *Schizymenia jonssonii*

## Abstract

North‐Atlantic records of *Schizymenia dubyi* extend along the eastern shores of the North Atlantic from Morocco to southern Britain and Ireland, and the species is also recorded from Iceland. A study was undertaken to confirm the identity of the specimens from Iceland that were geographically separate from the main distribution of *S. dubyi* and in contrast to other species of the genus did not have gland cells. We analyzed *rbc*L and COI molecular sequence data from Icelandic specimens and compared the results with those for *Schizymenia* specimens available in GenBank. For both markers, *Schizymenia* was shown to be a monophyletic genus. The Icelandic specimens were clearly genetically distinct from *S. dubyi* and formed a well‐supported clade with *Schizymenia* species from the Northern Pacific. Based on these results, we have described a new species, *Schizymenia jonssonii*, which can be distinguished by molecular phylogeny, its lack of gland cells and by being strictly intertidal. Crustose tetrasporophytes with identical COI and *rbc*L sequences were found at the same locations as foliose plants. *Schizymenia apoda* is reported for the first time in the UK, its identity confirmed by *rbc*L sequence data. In light of these findings, it is likely that by further molecular analysis of the genus *Schizymenia* in the north‐eastern Atlantic and the Mediterranean, a higher diversity of *Schizymenia* spp. will be discovered in this region.

**Abbreviations**
BP: before present
COI: Cytochrome c oxidase I gene
dNTP: deoxyribonucleotide triphosphate nucleobases

*Schizymenia* was erected by J. Agardh (1851) to accommodate several species of foliose red algae. However, due to difficulties in identifying foliose red algae, even at genus level, species have often been misidentified (Abbott 1967, DeCew et al. 1992). Indeed, many of the species earlier referred to *Schizymenia* have since been synonymized or transferred to other genera (Dawson 1961, Lindstrom 1985, 1986, Hansen 1989, Alongi and Cormaci 1993), and are now distributed among more than 10 different genera (Guiry and Guiry 2019). Species within the genus *Schizymenia* are very difficult to distinguish, and DeCew et al. (1992) in their studies failed to find any significant difference between the Atlantic species *S. dubyi*, the type species of the genus (Kylin 1932), and *S. pacifica* from the Pacific, in morphology, anatomy, or life history. These species have since been shown to belong to phylogenetically separate clades within the genus (Gabriel et al. 2011, Saunders et al. 2015).

Currently, there are 10 species recognized for the genus *Schizymenia* (Guiry and Guiry 2019). Some of these are rare and have hardly been recorded since their original description (Dawson 1961, Abbott 1967, Silva et al. 1996, Guiry and Guiry 2019). Recent molecular studies have shown the genus *Schizymenia* to be more diverse than previously thought. In the North Pacific, a new species, *Schizymenia tenuis*, was recently described and another undescribed species “*Schizymenia* sp._1Cal” recorded, based on molecular data (Saunders et al. 2015). A few more, yet undescribed species have also been found in New Zealand (Adams 1994, D'Archino and Zuccarello 2013, Nelson and Sutherland 2016).

Species of *Schizymenia* form a blade that can be several hundred millimeters long, contain gland cells, and bear cystocarps in the blade (Kylin 1932). Three species, *Schizymenia dubyi*,* S. pacifica,* and *S. novae‐zelandiae*, have been shown by cultural studies to have a life cycle with two dissimilar generations, an upright foliose gametophyte and a crustose tetrasporophyte identical to crusts previously known as *Haematocelis* (Ardré 1977, 1980, Sciuto et al. 1979, DeCew et al. 1992, Hawkes in Adams 1994). More species of the genus have been shown using molecular evidence to have two dissimilar life history phases (Saunders et al. 2015). All the species are found in the subtidal zone, some down to a depth of 30–55 m (Taylor 1945, Abbott 1967) and some are also found in the intertidal zone (e.g., Dixon and Irvine 1977, Saunders et al. 2015).

All known *Schizymenia* species are found in the Pacific, except *S. obliqua*, which has only been found in the Indian Ocean (Abbott 1967, Silva et al. 1996, Saunders et al. 2015). Two species, *S. dubyi* and *S. apoda* are also known from the Atlantic and the Indian Oceans (Silva et al. 1996, Gabriel et al. 2011).

In Iceland, *Schizymenia* was first registered by Caram and Jónsson (1972) in their Icelandic checklist as *Schizymenia dubyi*. Previously, H. Jónsson (1901) had recorded *Dilsea edulis* (=*D. carnosa*) from Iceland. However, this species has not been recorded in Iceland since and examination of H. Jonsson's specimens, kept in the Botanical Museum in Copenhagen (C), showed them to be a *Schizymenia* sp. On a visit in August 2007 to the collecting site of H. Jónsson in the intertidal zone at Öndverðarnes, *Schizymenia* sp. was found to be common (K. Gunnarsson, pers. obs.). The Icelandic specimens of *Schizymenia* sp. are structurally and anatomically similar to other *Schizymenia* species in all aspects except for the total lack of gland cells and that they are only found in the intertidal zone. “*Haematocelis”* **‐**like crusts have been found among the foliose *Schizymenia* at many sites in Iceland.

The present‐day distribution of *Schizymenia* in Iceland is from the southwest coast, where it is relatively common, becoming sparser along the West coast and into the middle part of the colder North coast. It is not found in the coldest, eastern part, of the coast. A similar pattern is encountered by species that have their main distribution area south of Iceland and their northern limit of distribution along the Icelandic coast (as e.g., *Chondrus crispus, Corallina officinalis*, and *Pelvetia canaliculata*). These species are common in the Faeroes, southern Scandinavia, and Scotland, whereas *Schizymenia*, except for unconfirmed records (due to a lack of specimens) from northern Scotland, is not found in these areas (Rueness 1977, Nielsen et al. 1995, Nielsen and Gunnarsson 2001, Hardy and Guiry 2003). The collecting sites for specimens of *Schizymenia* closest to Iceland in the North Atlantic are in southern England and southern Ireland.

The disjunct distribution of *Schizymenia* in the northern North Atlantic, the lack of gland cells in the specimens collected in Iceland, and the observation that they have only been found in the intertidal zone evoked suspicion that the *Schizymenia* found in Iceland might be genetically separated from the more southern relatives. In this study, we used COI and *rbc*L molecular markers to analyze phylogenetic relations within the genus *Schizymenia* with the aim of resolving the identity and affinity of the species found in Iceland.

## Materials and methods

### Sampling

*Schizymenia* plants used in the molecular analysis were collected at the localities listed in Table S1 in the Supporting Information. A piece of each specimen, c. 1–2 cm^2^_,_ was cut off and placed in silica gel for drying. Voucher specimens were dried on herbarium sheets and deposited in the herbarium of the Icelandic Institute of Natural History (ICEL). Herbarium abbreviations follow Thiers (2018).

A sampling location at Stekkjarvikur, Reykjanes peninsula, southwestern Iceland (64°1.770′ N, 22°14.616′ W), was visited every month at low water, spring tide from April 2016 to May 2017 to monitor seasonal changes in morphology and reproduction in both the foliose and crustose plants of *Schizymenia*. Plants were collected to examine the presence of reproductive structures, and the lengths of the ten largest foliose specimens were measured. The substratum at the study site is of uneven, basaltic lava rocks. The mean tidal range at spring tide is c. 3.5 m and the sea surface temperatures vary on average from 1.5°C for March to c. 13.5°C in August (Icelandic Coast Guard 2019, Marine and Freshwater Research Institute 2019).

In the laboratory, the plants were photographed and hand‐sectioned for microscopic observations using a Leitz RMZ light microscope equipped with a Leitz DFC320 digital microphotographic camera. Voucher specimens were prepared and deposited at the Natural History Institute in Reykjavik (ICEL).

### DNA extraction and sequencing

Pieces c. 5–10 mm^2^ of silica‐dried *Schizymenia* blades and crusts were ground in a mortar with chemically pure sand (Merck, Darmstad, Germany) to a fine powder. The powder was rehydrated in CTAB (cetyltrimethylammonium bromide), Sarkosyl, and proteinase K (500:50:10) and then DNA was dissolved with SEVAC (chloroform:isoamyl alcohol, 24:1) at 65°C and precipitated with isopropanol and ethanol. The DNA was then cleaned using GFx DNA cleaning kit (GE Healthcare, UK) following the manufacturer's protocol. To each 1 μL DNA sample, we added 22 μL of PCR mixture contained, 2.5 μL buffer, 1.5 μL MgCl_2_, 0.5 μL dNTP, 1 μL of each forward and reverse primers, and 0.5 μL Taq (Biotaq DNA Polymerase kit; Bioline Ltd, UK) for amplification. The plastid‐encoded *rbc*L was amplified using F57 and R1442 primers (Freshwater and Rueness 1994). The mitochondrial COI gene was amplified using GAZF1 and GAZR1 primers (Saunders 2005) and M131F and M13Rx (Saunders and Moore 2013). PCR amplifications were undertaken in a Techne thermal cycler (Cole‐Parmer Ltd., St. Neots, UK). For *rbc*L amplification, we used an initial denaturation step at 95°C for 2 min, followed by 35 cycles of denaturation at 95°C for 1 min, annealing at 47°C for 1 min and extension at 72°C for 2 min, and then a final extension at 72°C for 2 min. For CO1, we used an initial denaturation step at 94°C for 2 min, followed by five cycles of denaturation at 95°C for 30 s, annealing at 45°C for 30 s and 1 min extension at 72°C and then 35 cycles starting with denaturation at 94°C for 30 s and annealing at 46.5°C for 30 s, and an extension at 72°C finishing by a final extension at 72°C for 1 min (Saunders and Moore 2013). The amplification success was tested by agarose gel electrophoresis. The PCR amplifications were undertaken with 18 samples of foliose *Schizymenia* (17 from Iceland and 1 from UK) and 3 samples of “*Haematocelis*”‐like crusts. The PCR products were sent for DNA sequencing at the sequencing laboratory of the Natural History Museum in London on an ABI 3730 XL capillary DNA analyser (Applied Biosystems).

### Phylogenetic analysis

The resulting sequences were edited with BioEdit, version 7.2.6 (Hall 1999). Sequences from GenBank of *rbc*L and COI for *Schizymenia* spp. were compared with our sequences and *Platoma cyclocolpum* and *Titanophora weberae* used as outgroup (Saunders et al. 2015). Information on GenBank accession numbers, origin, date of collection, etc., of the sequences used to construct the phylogenetic trees is shown in Table S1.

Phylogenetic analyses were performed with maximum likelihood and Bayesian methods. Maximum likelihood (ML) analyses were done using MEGA5 (Tamura et al. 2011). Support for ML analysis was assessed both with approximate likelihood ratio test and bootstrap values were created with 1,000 resampling replicates for both *rbc*L and COI data. Bayesian analysis was conducted with MrBayes v3.2.6 (Ronquist et al. 2011) using a general time reversible model with invariable sites and gamma distribution (GTR+I+Γ) to describe the nucleotide sequence evolution, which was assessed as a good candidate by JModelTest2 with the Akaike Information Criterion (Guindon and Gascuel 2003, Darriba et al. 2012). This model was used in Bayesian analysis for both *rbc*L and COI. Markov chain Monte Carlo analysis was run for 1,000,000 generations with sampling every 1000 generations. Potential scale reduction factor scores were used to determine the number of generations run. The sump and sumt functions of MrBayes were used to calculate Bayesian posterior probability values. The first 25% of the samples from the cold chain were discarded as burn‐in. Maximum likelihood and bootstrap values were created using MEGA5 (Tamura et al. 2011).

## Results

### Taxonomy

**Figure 1:**
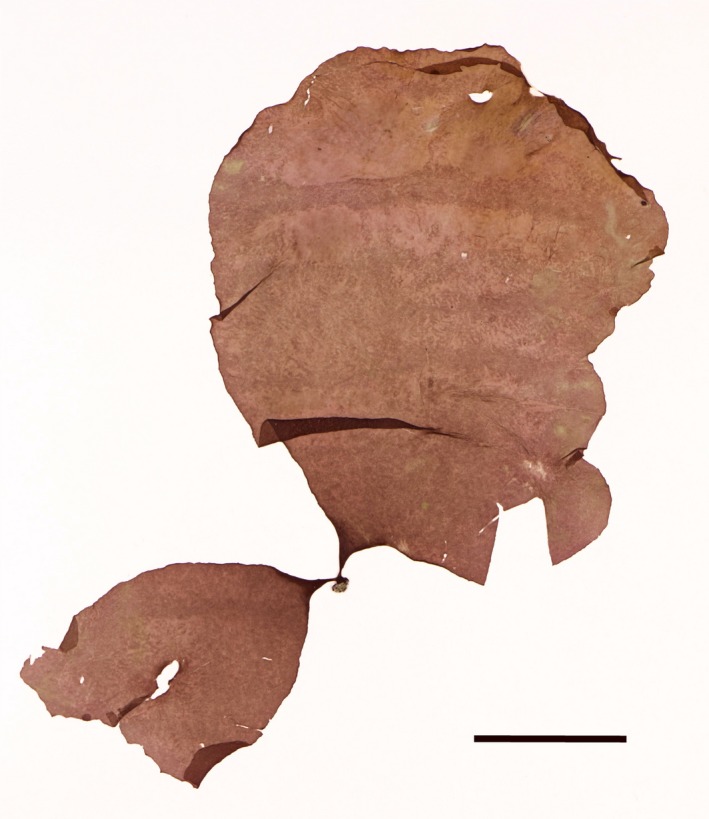
Holotype specimen of *Schizymenia jonssonii* sp. nov. (BM013844101), collected at Stekkjarvikur, SW Iceland on April 21, 2016. Scale = 2 cm. [Color figure can be viewed at http://www.wileyonlinelibrary.com]

***Schizymenia jonssonii*** K.Gunnarsson & J.Brodie sp. nov. (Fig. 1)

Diagnosis: Gametophytic phase foliose with surface either flattened or with irregular ridges and depressions. Thallus lacking gland cells. Color dark red to brownish red. Tetrasporophytic phase crustose, with uneven surface having numerous small swellings. Can equally be distinguished by its nucleotide sequences of the mitochondrial COI and plastid *rbc*L genes.

Description: Species consisting of an upright foliose phase and a “*Haematocelis”* crustose phase. Upright fronds have a cylindrical stipe, 1–3 mm long, and are attached with a discoid or slightly conical holdfast. Thallus oblong, sometimes lobed or split, 50–350 mm in length, semi‐gelatinous; surface either flattened or with irregular ridges and depressions. Color dark red to brownish red. Fronds are 250–600 μm thick (older blades in winter up to 900 μm).

**Figure 2:**
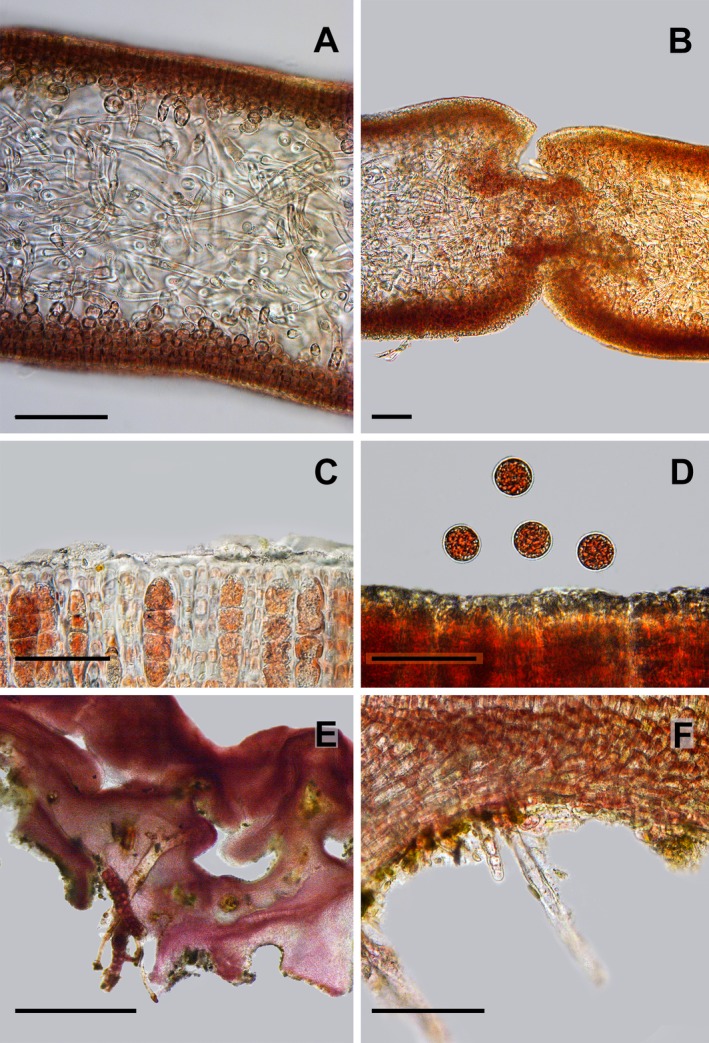
*Schizymenia jonssonii* sp. nov. (a) Transverse section (T.S.) of blade. (b) Cystocarps between the inner cortex and medulla in T.S. of the blade. Ostioles opening to the surface. (c) Zonate tetrasporangia just below the surface of a sporophyte crust. (d) Tetraspores being released. (e) T.S. of a tetrasporophyte with openings between layers. (f) Rhizoids on the underside of the tetrasporophyte. Scale (a–d and f) = 50 μm and (e) = 1 mm. [Color figure can be viewed at http://www.wileyonlinelibrary.com]

In surface view, cells are rounded, 7–9 μm in diameter. The blade consists of an inner medulla and an outer cortex (Fig. 2a). In transverse section (TS), the medulla consists of loosely interwoven, branched filaments of elongated cells, 3–7 μm in diameter, and up to more than 150 μm long. The cortex consists of cell rows, typically 6–8 cells (–20) in length in TS with dichotomous branching. The innermost cortical cells are spherical, 30 μm in diameter in TS. Cells diminish in size toward the surface and the outermost cortical cells are elongate, c. 7.5 × 15 μm in TS. The medullary filaments are connected by pit connections to the cortical branches but no secondary pit connections were observed. Gland cells are absent.

Cystocarps are visible as small dark‐red spots on the surface of the fertile blades and are most common in the distal end but are also occasionally found in the middle and the lower part of the thallus. Cystocarps are situated in the inner cortex or in between the medullary filaments, close to the cortex, 130–300 μm across and are without pericarp. Ostioles are visible as depressions in the surface of the thallus by the cystocarps (Fig. 2b). Individual carposporangia are 15–40 μm in diameter. Carpogonia and spermatangia were not observed.

Crusts are dark red in color, irregularly shaped, 1–7 cm across, and cartilaginous. Crusts are up to 2.5 mm thick and layered with darker and lighter bands seen in cross section. Horizontal basal filaments curve up and give rise to upright, narrower filaments. Tetrasporangia are zonate, produced in the surface layer, 50–70 μm × 15–20 μm (Fig. 2c). Released tetraspores are spherical, *c*. 25 μm in diameter (Fig. 2d). Surface of crust is uneven with numerous small swellings (Fig. 2e). Rhizoids, two to five cells long, are occasionally found on the underside of the crust (Fig. 2f).

Holotype: Gametophyte collected in the lower intertidal zone at Stekkjarvikur, Reykjanesskagi, Iceland (64°1.77′ N, 22°14.62′ W), April 21, 2016 (Fig. 1). GenBank accession numbers: *rbc*L = MN567259, COI = MN567252. Specimen deposited in the Cryptogamic Herbarium of the Natural History Museum, London (BM). BM museum number: BM013844101.

Isotype: Crustose tetrasporophyte, collected in the lower intertidal zone at Stekkjarvikur, Reykjanesskagi, Iceland (64°1.77′ N, 22°14.62′ W), April 21, 2016. GenBank accession numbers: *rbc*L = MN567260, COI = MN567253, deposited in the Cryptogamic Herbarium of the Natural History Museum, London (BM). BM Museum number: BM013844102.

Etymology: The species is named in honor of the Icelandic phycologist Sigurður Jónsson, for his contribution to the knowledge of Icelandic macroalgae and phycology in general.

Paratypes: Gametophytes collected at Sölvaflá, Vestmannaeyjar, August 12, 1999 (Voucher =ICEL586), Stekkjarvikur, Reykjanesskagi, April 21, 2016 (Voucher =ICEL13226), Öndverðarnes, August 8, 2007 (Voucher =ICEL6225), Flatey, Breidifjördur, June 17, 1977 (Voucher =ICEL856), Skálavík, July 3, 2008 (Voucher =ICEL7748) and Laugatangi, Hrísey, June 14, 2006 (Voucher =ICEL5343). All paratype specimens are deposited in the algal herbarium of the Icelandic Institute of Natural History (ICEL).

Type Locality: Stekkjarvikur, Reykjanesskagi, Iceland (64°1.77′ N, 22°14.62′ W).

**Figure 3:**
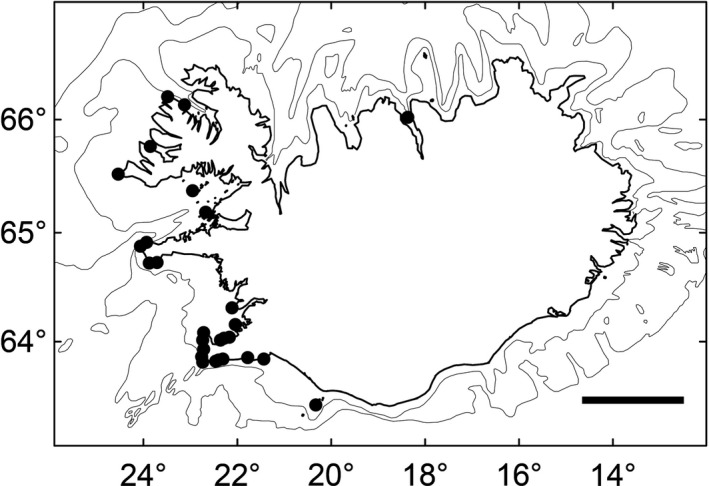
Distribution of *Schizymenia jonssonii* sp. nov. (black circles). Depth contours for 100 and 200 m are shown. Scale = 100 km.

Distribution: *Schizymenia* is found in Vestmannaeyjar Archipelago off the south coast of Iceland and is distributed more or less continuously from there, along the southwest coast. It is then found sporadically along the west and the northwest coast and at one locality on the north coast (Fig. 3).

**Figure 4:**
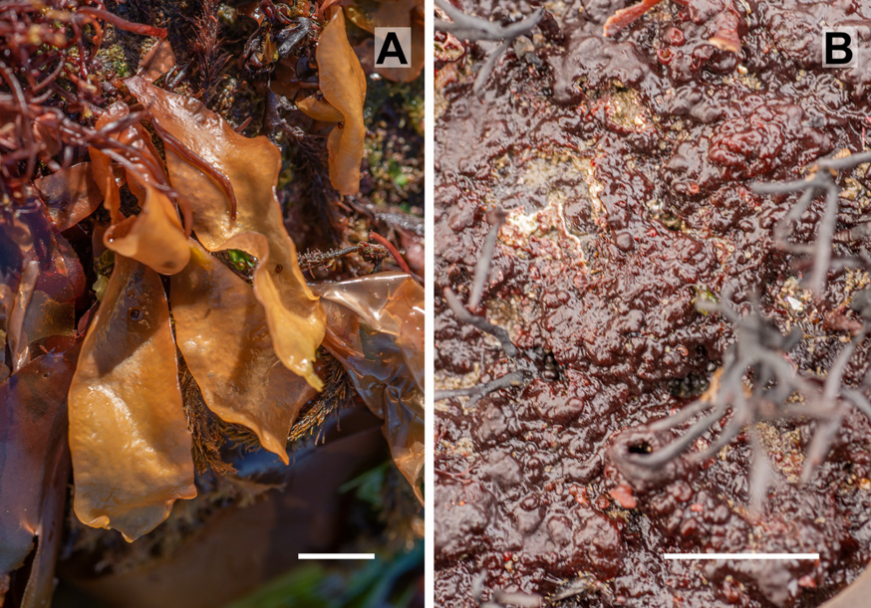
Life history phases of *Schizymenia jonssonii* sp. nov. growing in the lower intertidal in Stekkjarvikur, SW‐Iceland. (a) Blades – gametophyte phase, July 4, 2016. (b) Crusts – tetrasporophyte phase, March 1, 2018. Scale = 2 cm. [Color figure can be viewed at http://www.wileyonlinelibrary.com]

The upright fronds started to appear in February, most often attached directly to the rock by a small (2–4 mm diameter), conical holdfast, but sometimes they grew up from a *Haematocelis*‐like crust. The fronds increased in size until July when they were at their maximum size. From May until August, the fronds were mostly yellow in color (Fig. 4a) and were often seen with bleached spots. During the period from June to August, the blades had epiphytes and were grazed by numerous gastropods. In September, the blades started to erode and many of the plants died. Only a very few, small plants were observed from December to February. Cystocarps were first observed in September and last seen in March in some of the small blades that had over‐wintered from the previous year.

Crusts were cartilaginous, with a firm, uneven surface and often with dark red swellings, 1–5 cm in diameter (Fig. 4b). The crusts were loosely attached to the substratum and were easily detached. During the winter, the crusts were without overgrowth of ephemeral algae and could be spotted by eye due to their slightly lighter color than other red algal crusts, *Hildenbrandia* spp. and *Haemescharia hennedyi* that were growing in the vicinity. In the summer, the crusts were overgrown by the red and green algae *Cystoclonium purpureum*,* Ceramium virgatum*, and *Ulva fenestrata* (cf Hughey et al. 2019) and difficult to spot. Crusts were up to 7 cm in diameter. Tetrasporangia were first seen in December in the inner layers of some older crusts. In January, tetrasporangia were observed in the surface layer on many of the crusts but often the sporangia contained only two spores. The portion of sporangia with four spores increased from January to March (Fig. 2c). In April, only a few sporangia were observed, all with two spores. The older crusts were layered, and often had holes both between layers and between the crust and the substratum (Fig. 2e). These holes were inhabited by a variety of invertebrates. The crustose plants grew along with the blade‐phase on moderately exposed to exposed shores and were found throughout the year.

*Phylogenetic analysis*. 17 *rbc*L sequences of 1210 bp and 17 COI sequences of 680 bp were obtained from specimens of *Schizymenia jonssonii* collected in Iceland. An additional *rbc*L sequence was obtained for a specimen collected in Plymouth, UK. All the sequences from the Icelandic samples turned out to be identical for the respective markers. The ML tree for *rbc*L contained 23 *Schizymenia* sequences of which 6 were new sequences from Icelandic specimens and one from Plymouth.

**Figure 5:**
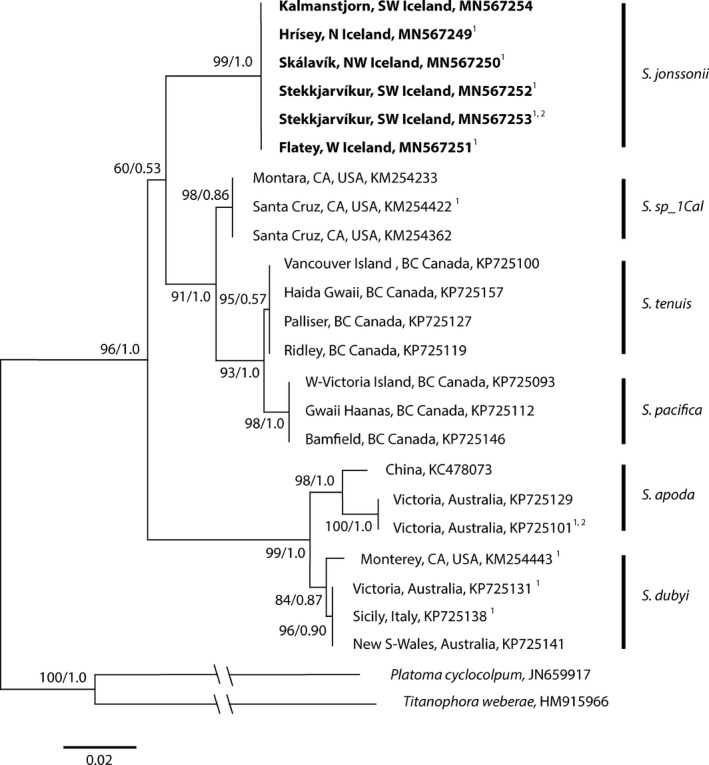
Maximum likelihood tree for *Schizymenia* spp. drawn using COI marker with *Platoma cyclocolpum* and *Titanophora weberae* as outgroup. GenBank accession numbers are given for each sequence. Values at nodes: ML bootstrap values/Bayesian posterior probability. Sequences produced in the present study are in bold. Scale bar = 0.02 substitutions per site. ^1^Specimen has also been sequenced for *rbc*L and is shown in Fig. 6. ^2^Tetrasporophyte. For details on the specimens, from which the sequences were obtained, see supplementary data in Table S1.

**Figure 6:**
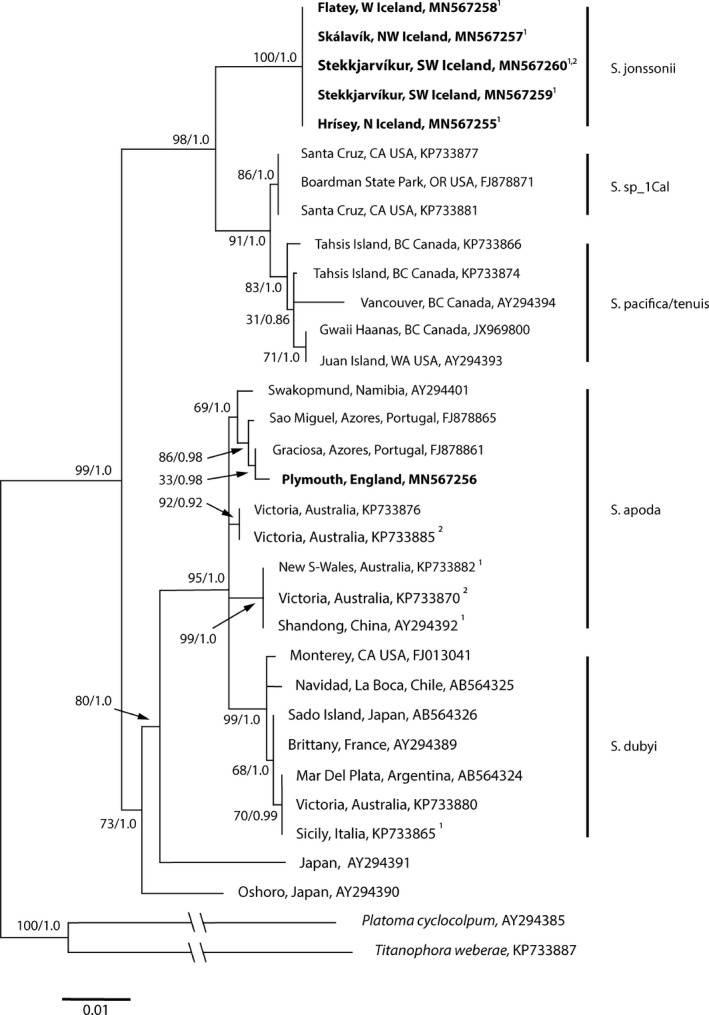
Maximum likelihood tree for *Schizymenia* spp. using *rbc*L sequences with *Platoma cyclocolpum* and *Titanophora weberae* as outgroup. Values at nodes: ML bootstrap values/Bayesian posterior probability. Sequences produced in the present study are in bold. Scale bar = 0.01 substitutions per site. ^1^Specimen has also been sequenced for COI and is shown in Fig. 5. ^2^Tetrasporophyte. For details on the specimens, from which the sequences were obtained, see supplementary data in Table S1.

Analysis of COI and *rbc*L sequences of *Schizymenia jonssonii,* and *Schizymenia* sequences available from GenBank showed a monophyletic tree and two distinct clades of species (Figs. 5 and 6). The new species *S. jonssonii* formed a highly supported cluster with the Pacific species *S. pacifica*,* S. tenuis* and the undescribed species “*Schizymenia*. sp._1Cal” (Saunders et al. 2015), and was most closely related to “*Schizymenia* sp._1Cal” with c. 97–98% similarity for the two markers studied. In the phylogenetic tree, this clade was distinctly separated from a clade containing the two other species of *Schizymenia* found in the Atlantic, *S. dubyi,* and *S. apoda*. The *rbc*L sequences from two specimens of unidentified *Schizymenia* spp. from Japan, clustered with *S. dubyi* and *S. apoda*, but formed separate branches. The *rbc*L sequence of a sample of *Schizymenia* sp. from Plymouth turned out to be conspecific with *S. apoda* from Namibia and the Azores (Fig. 5). The differences in *rbc*L and COI sequences between the two clades ranged from 4.6% to 4.8% and 5.1% and 6.8%, respectively (Table 1).

**Table 1:** Percentage interspecific differences in *rbc*L/COI sequences among species of *Schizymenia*

	*S. jonssonii*	*S*. sp. 1Cal	*S. tenuis*	*S. pacifica*	*S. dubyi*
*S*. sp. 1Cal	2.33/3.48				
*S. tenuis*	2.56/4.09	0.45/1.67			
*S. pacifica*	2.71/4.24	0.38/2.12	0.23/0.76		
*S. dubyi*	4.44/5.91	4.66/5.15	4.66/5.30	4.81/5.54	
*S. apoda*	4.51/6.82	4.36/6.06	4.51/6.21	4.66/6.36	1.43/2.27

## Discussion

The results from the present analysis of COI and *rbc*L data reveal a new species of *Schizymenia* in the Atlantic, *S. jonssonii* K.Gunnarsson & J.Brodie, which adds a third species of *Schizymenia* to the North Atlantic seaweed flora. This species differs from other *Schizymenia* species by being strictly intertidal and lacking gland cells. It is only distantly related to the other species of the genus found in the Atlantic (i.e., *S. dubyi* and *S. apoda*) but clusters more closely with the Pacific species *S. tenuis*,* S. pacifica* and the undescribed species “*Schizymenia* sp._1Cal” (Saunders et al. 2015).

Analysis of the *rbc*L marker from a specimen collected in Plymouth proved to be conspecific with *Schizymenia apoda* from the Azores and Namibia. This is the first record of *S. apoda* in the UK and the first genetic sequence analysis of the genus *Schizymenia* from the UK.

The uncertainty of the identity of the species in relation to the type specimen of type of the genus, *Schizymenia dubyi,* which is from Cherbourg, France, cannot be resolved until sequence data of the type specimen is available. We attempted without success to obtain a sequence from the type specimen of the genus dated 1826 (material kindly given to us by the University of Strasbourg Herbarium [STR]) following the method and recommendations given by Hughey and Gabrielson (2012). Although there is a DNA sequence from a specimen identified as *S. dubyi* for the west of Brittany, the presence of species identified as *S. apoda* nearby makes it possible that either of the two species or even another species will prove to be identical to the type specimen. This is further confounded by possible introgression of *S. apoda* and *S. dubyi* (Saunders et al. 2015). The clear genetic separation of the two clades in the phylogenetic tree (Figs. 5 and 6) and the high sequence differences between the two clades (Table 1) makes an argument for considering a revision of the concept of the genus *Schizymenia,* including the creation of a new genus, but until more species of the genus have been sequenced and the phylogenetic position of the type has been established, this remains a future goal.

*Schizymenia dubyi* has been recorded from Morocco (Benhissoune et al. 2003), in the Mediterranean sea (Sciuto et al. 1979, Alongi and Cormaci 1993) and along the Atlantic coasts of Portugal (Ardré 1970), Northern Spain (Rodriguez and Moliner 2010), and France (Gayral 1966) north to the UK (Hardy and Guiry 2003) as well as Iceland (Gunnarsson and Jónsson 2002). The Icelandic specimens have now been shown to belong to a separate species and apart from the *S. apoda* sequences from the Azores, only one *rbc*L sequence is available in GenBank of *Schizymenia* from the Atlantic coast of Europe. Discovering the new species *S. jonssonii* in Iceland and finding *S. apoda* in the Plymouth area in Britain emphasizes the need for further molecular studies on *Schizymenia* specimens from the area to establish the identity and distribution of *Schizymenia* spp. in the North Atlantic and the Mediterranean Sea.

Both *Schizymenia apoda* and *S. dubyi* have been found at several sites in the Pacific (Hughey and Miller 2009, Gabriel et al. 2011, Kim et al. 2012, D'Archino and Zuccarello 2013, Saunders et al. 2015), and *S. dubyi* has also been found on the Atlantic coast of Argentina (Ramirez et al. 2012). In some cases, they are thought to have been recently introduced (Ramirez et al. 2012, D'Archino and Zuccarello 2013).

As there are still five additional species of *Schizymenia* listed in Algaebase (Guiry and Guiry 2019) for which there are no sequence data yet, the question remains as to whether one of them might be synonymous with *S. jonssonii*. Two of them, *S. violacea* and *S. johnstonii* (Setchell and Gardner 1924) from the Gulf of California are considered to be synonymous and belong to the genus *Grateloupia*, as *G. violacea* (Dawson 1944, 1961, Gabrielson et al. 2019). *Schizymenia obliqua* was found on the island St Paul in the Indian Ocean and is only known from its original collection by Grunow (1868, Silva et al. 1996). This species was first described as a variety of *S. erosa*, differing “just” in the form of the blade that also resembled *Iridaea curvata* (Grunow 1868). Kylin (1932) found that the original specimen of *S. erosa* was a male plant belonging to the genus *Iridaea*. *Schizymenia ecuadoreana* is found in the Galapagos Islands. It is a deep‐water species with distinctive gland cells (Taylor 1945, Abbott 1967). *Schizymenia binderi* is found on the Pacific coast of South America (Papenfuss 1964, Ramírez and Santelices 1991) and also possesses gland cells (Kylin 1932). Considering the strict subtidal habitat of the two previously named species, with plants growing to a depth of 55 m (Taylor 1945), distinct gland cells and their distribution in the southern hemisphere, they are unlikely to be synonymous with, or the source of the *Schizymenia* population in Iceland.

The DNA sequence analysis shows a clear separation of two phylogenetically distinct species groups of the genus *Schizymenia*, one represented by the species *S. dubyi* and *S. apoda* and the other with the closely related species *S. pacifica*,* S*. *tenuis*, “*Schizymenia*. sp._1Cal,” and the new species *S. jonssonii* (Figs. 5 and 6). This indicates a firm and extended geographical isolation of the two clades, perhaps, between the Atlantic and the Pacific and that *S. jonssonii* has arrived in the Atlantic after the evolutionary separation of the clades and that *S. apoda* and *S. dubyi* are possibly inversely, relatively recent introductions into the Pacific.

The results raise the question of how old the introduction and the genetic separation of *Schizymenia jonssonii* from its Pacific relatives is likely to be. *Schizymenia jonssonii* possibly has an ancestor of Pacific origin and colonized the Atlantic after the opening of the Bering Strait, evolved in the Atlantic and is a relic from the last glacial maximum (LGM). But it is equally possible that it is a relatively recent introduction into the Atlantic. The following two scenarios could possibly explain the presence of the new species in Iceland. **A**: The species passed through the Bering Strait during ancient warm periods after 3.5 million years BP and colonized the North Atlantic. The opening of the Bering Strait is dated at about 5.5 million years BP (Gladenkov et al. 2002) while inflow of biota from the Pacific into the Atlantic possibly started much later or at about 3.5 million years BP (Vermeij 1991). The Icelandic coast is thought to have been completely covered by ice during the LGM in the North Atlantic, from 26,500 years BP to at least about 14,600 to 12,700 years BP (Ingólfsson et al. 2010). If the species is to have survived in the Atlantic for a more extended period, it would have been pushed southwards by the glaciation at least to the English Channel (Sejrup et al. 2005, Carr et al. 2006) and when the glacier started retreating it would have moved toward the Arctic in the wake of the deglaciation. If this was the case, it would be expected for there to be traces of the species left behind south of Iceland (e.g., in the Faroes, southern Scandinavia or the UK). However, since then it appears to have died out south of Iceland and in the Pacific (or the species is still living cryptically further south in Europe and/or in the Pacific). **B:** The species colonized Iceland from the Pacific after LGM. The source population in the Pacific died out (or a cryptic source population still exists in the Pacific). Only more molecular studies of *Schizymenia* from more locations in the North Pacific will help solve this puzzle.

Until now only *Schizymenia dubyi* had been recorded for Britain and Ireland. The finding of *S. apoda* in England raises the question of the identity of *Schizymenia* in the region. To resolve this problem, more sequence data are needed from contemporary and historical material. This has been proved to be valuable for the study of algal diversity (this study and Gunnarsson et al. 2016).

Considering the discovery of the new species in Iceland, the presence of *Schizymenia apoda* in England, and the extreme difficulty in distinguishing species within the genus, it is likely that there is greater diversity of *Schizymenia* spp. at the local level in the north‐eastern Atlantic.

The authors thank Svanhildur Egilsdóttir for assistance in the field and preparing the photos. Chris Williamson and Li‐En Yang are thanked for help with laboratory work and data analysis and Jo Wilbraham, senior algal curator, Natural History Museum, for assisting with herbarium specimens.

## Conflicts of interest

The authors declare no conflicts of interest.

## Supporting information


**Table S1**. GenBank accession‐numbers, species, voucher identity, sampling localities, collectors and collecting dates for specimens used in molecular phylogenetic analysis. Sequences that were generated in the present study are in bold.

## References

[jpy12957-bib-0001] Abbott, I. A. 1967 Studies in some foliose red algae of the Pacific coast II. *Schizymenia* . Bull. So. Calif. Acad. Sci. 66:161–74.

[jpy12957-bib-0002] Adams, N. M. 1994 Seaweeds of New Zealand. Canterbury University Press, Christchurch, NZ, 360 pp.

[jpy12957-bib-0003] Agardh, J. G. 1851 Species genera et ordines algarum, seu descriptiones succinctæ specierum, generum et ordinum, quibus algarum regnum constituitur. Volumen secundum: algas florideas complectens. Part 1. C.W.K. Gleerup, Lund, pp. i–xii, 1–351.

[jpy12957-bib-0004] Alongi, G. & Cormaci, M. 1993 Un comportement particulier du *Schizymenia dubyi* de Sicile, et interpretation du cas du *Schizymenia epiphytica* (Gigartinales, Rhodophyceae). Cryptogam. Algol. 14:173–81.

[jpy12957-bib-0005] Ardré, F. 1970 Contribution à l’étude des algues marines du Portugal. I. La Flore. Portugaliae Acta Biol. B. 10:137–556.

[jpy12957-bib-0006] Ardré, F. 1977 Sur le cycle de *Schizymenia dubyi* (Chauvin ex Duby) J. Agardh (Némastomacée, Gigartinale). Rev. Algol. N. S. 12:73–86.

[jpy12957-bib-0007] Ardré, F. 1980 Observations sur le cycle de développement du *Schizymenia dubyi* (Rhodophycée, Gigartinale) en culture, et remarques sur certains genres de Némastomacées. Cryptogam. Algol. 1:111–40.

[jpy12957-bib-0008] Benhissoune, S. , Boudouresque, C. F. , Perret‐Boudouresque, M. & Verlaque, M. 2003 A checklist of the seaweeds of the Mediterranean and Atlantic coasts of Morocco. IV. Rhodophyceae ‐ Ceramiales. Bot. Mar. 46:55–68.

[jpy12957-bib-0009] Caram, B. & Jónsson, S. 1972 Nouvel inventaire des algues marines de l'Islande. Acta Bot. Isl. 1:5–31.

[jpy12957-bib-0010] Carr, S. J. , van der Holmes, R. , Meer, J. J. M. & Rose, J. 2006 The last glacial maximum in the North Sea basin: Micromorphological evidence of extensive glaciation. J. Quaternary Sci. 21:131–53.

[jpy12957-bib-0011] D'Archino, R. & Zuccarello, G. C. 2013 First record of *Schizymenia apoda* (Schizymeniaceae, Rhodophyta) in New Zealand. N. Z. J. Mar. Freshw. Res. 48:155–62.

[jpy12957-bib-0012] Darriba, D. , Taboada, G. L. , Doallo, R. & Posada, D. 2012 jModelTest 2: more models, new heuristics and parallel computing. Nat. Methods 9:772. 10.1038/nmeth.2109 PMC4594756 22847109

[jpy12957-bib-0013] Dawson, E. Y. 1944 The marine algae of the Gulf of California. A. Hancock Pacific Exped. 3:1–450.

[jpy12957-bib-0014] Dawson, E. Y. 1961 Marine red algae of Pacific Mexico. Part 4. Gigartinales. Pac. Nat. 2:191–343.

[jpy12957-bib-0015] DeCew, T. C. , Silva, P. C. & West, J. A. 1992 Culture studies on the relationship between *Schizymenia* and *Haematocelis* (Gigartinales, Rhodophyceae) from the Pacific coast of North America. J. Phycol. 28:558–66.

[jpy12957-bib-0016] Dixon, P. S. & Irvine, L. M. 1977 Seaweeds of the British Isles. Volume 1 Rhodophyta. Part 1. Introduction, Nemaliales, Gigartinales. British Museum (Natural History), London, 252 pp.

[jpy12957-bib-0017] Freshwater, D. W. & Rueness, J. 1994 Phylogenetic relationships of some European *Gelidium* (Gelidiales, Rhodophyta) species, based on *rbc*L nucleotide sequence analysis. Phycologia 33:187–94.

[jpy12957-bib-0018] Gabriel, D. , Schills, T. , Parente, I. P. , Draisma, S. G. A. , Neto, A. I. & Fredericq, S. 2011 Taxonomic studies in the Schizymeniaceae (Nemastomatales, Rhodophyta): on the identity of *Schizymenia* sp. in the Azores and the generic placement of *Nemastoma confusum* . Phycologia 50:109–21.

[jpy12957-bib-0019] Gabrielson, P. in Guiry, M. D. & Guiry, G. M. 2019 AlgaeBase. World‐wide electronic publication, National University of Ireland, Galway http://www.algaebase.org; searched on 23 July 2019.

[jpy12957-bib-0020] Gayral, P. 1966 Les Algues Des Côtes Françaises (Manche & Atlantique); notions fondamentales sur l’écologie, la biologie et la systématique des algues marines. ed. Ouest‐France, 220 pp.

[jpy12957-bib-0021] Gladenkov, A. Y. , Oleinik, A. E. , Marincovich, L. Jr & Barinov, K. B. 2002 A refined age for the earliest opening of Bering Strait. Paleogeogr. Paleoclimatol. Paleoecol. 183:321–8.

[jpy12957-bib-0022] Grunow, A. 1868 Reise der österreichischen Fregatte Novara um die Erde in den Jahren 1857, 1858, 1859 unter den Befehlen des Commodore B. von Wüllerstorf‐Urbair. Botanischer Theil. Erster Band. Sporenpflanzen. Wien: Aus der Kaiserlich Königlichen Hof‐ und Staatsdruckeri in Commission bei Karl Gerold's Sohn, 404 pp.

[jpy12957-bib-0023] Guindon, S. & Gascuel, O. 2003 A simple, fast and accurate method to estimate large phylogenies by maximum‐likelihood. Systematic Biol. 52:696–704. 10.1080/10635150390235520 14530136

[jpy12957-bib-0024] Guiry, M. D. & Guiry, G. M. 2019 AlgaeBase. World‐wide electronic publication, National University of Ireland, Galway http://www.algaebase.org; searched on 20 April 2019.

[jpy12957-bib-0025] Gunnarsson, K. , Egilsdóttir, S. , Nielsen, R. & Brodie, J. 2016 A collection‐based approach to the species and their distribution based on the bladed Bangiales (Rhodophyta) of Iceland. Bot. Mar. 59:223–9.

[jpy12957-bib-0026] Gunnarsson, K. & Jónsson, S. 2002 Benthic marine algae of Iceland: revised checklist. Cryptogam. Algol. 23:131–58.

[jpy12957-bib-0027] Hall, T. A. 1999 BioEdit: a user‐friendly biological sequence alignment editor and analysis program for Windows 95/98/NT. Nucleic Acids Symp. Ser. 41:95–8.

[jpy12957-bib-0028] Hansen, G. I. 1989 *Schizymenia dawsonii* and its relation to the genus *Sebdenia* (Sebdeniaceae, Rhodophyta). Taxon 38:54–9.

[jpy12957-bib-0029] Hardy, G. & Guiry, M. D. 2003 A Check‐list and Atlas of the Seaweeds of Britain and Ireland. British Phycological Society, London, 437 pp.

[jpy12957-bib-0030] Hughey, J. R. & Gabrielson, P. W. 2012 Comment on “Acquiring DNA sequence data from dried archival red algae (Florideophyceae) for the purpose of applying available names to contemporary genetic species: a critical assessment”. Botany 90:1191–4.

[jpy12957-bib-0031] Hughey, J. R. , Maggs, C. A. , Mineur, F. , Jarvis, C. , Miller, K. A. , Shabaka, S. H. & Gabrielson, P. W. 2019 Genetic analysis of the Linnaean *Ulva lactuca* (Ulvales, Chlorophyta) holotype and related type specimens reveals name misapplications, unexpected origins, and new synonymies. J. Phycol. 55:503–8. 30907438 10.1111/jpy.12860

[jpy12957-bib-0032] Hughey, J. R. & Miller, K. A. 2009 Noteworthy collection: *Schizymenia dubyi* (Chauvin ex Duby) J. Agardh, Rhodophyta, new to California. Madroño 56:64.

[jpy12957-bib-0033] Icelandic Coast Guard 2019 Tide tables 2019. Hydrographic Department, Reykjavik, 27 pp.

[jpy12957-bib-0034] Ingólfsson, O. , Norðdal, H. & Schomacher, A. 2010 Deglaciation and holocene glacial history of Iceland. Dev. Quat. Sci. 13:51–68.

[jpy12957-bib-0035] Jónsson, H. 1901 The marine algal vegetation of Iceland. 1. Rhodophyceae. Bot. Tidsskr. 24:127–55.

[jpy12957-bib-0036] Kim, S. Y. , Seo, T. H. , Park, J. K. , Boo, G. H. , Kim, K. M. & Boo, S. M. 2012 *Cryptonemia rotunda* (Halymeniales) and *Schizymenia apoda* (Nemastomatales), two new records of red algae from Korea. Algae 27:1–8.

[jpy12957-bib-0037] Kylin, H. 1932 Die Florideenordung Gigartinales. Lunds Univ. Årsskr. N.F. Avd. 2. 28:1–88.

[jpy12957-bib-0038] Lindstrom, S. 1985 Nomenclatural and taxonomic notes on *Dilsea* and *Neodilsea* (Dumontiaceae, Rhodophyta). Taxon 34:260–6.

[jpy12957-bib-0039] Lindstrom, S. 1986 Nomenclatural and taxonomic notes on species of the red algal genera *Halymenia* (Cryptonemiaceae) and *Weeksia* (Dumontiaceae). Taxon 35:531–3.

[jpy12957-bib-0040] Marine and Freshwater Research Institute , 2019 Sea surface temperatures at the Icelandic coast. http://www.hafro.is/Sjora/, searched on 26 April 2019.

[jpy12957-bib-0041] Nelson, W. A. & Sutherland, J. E. 2016 *Predaea rosa* sp. nov (Nemastomatales, Rhodophyta): a cool‐temperate species from southern New Zealand. Phycologia 56:167–75.

[jpy12957-bib-0042] Nielsen, R. & Gunnarsson, K. 2001 Seaweeds of the Faroe Islands. An annotated checklist. Fróðskaparritið 49:45–108.

[jpy12957-bib-0043] Nielsen, R. , Kristiansen, A. , Mathiesen, L. & Mathiesen, H. 1995 Distributional index of the benthic macroalgae of the Baltic Sea area. Acta Bot. Fenn. 155:1–55.

[jpy12957-bib-0044] Papenfuss, G. F. 1964 Catalogue and bibliography of Antarctic and Sub‐Antarctic benthic marine algae *In* Lee M. O. [Ed.] Bibliography of the Antarctic Seas, Vol 1 American Geophysical Union, Washington, DC, pp. 1–76.

[jpy12957-bib-0045] Ramirez, M. E. , Nuñez, J. D. , Ocampo, E. H. , Matula, C. V. , Suzuki, M. , Hashimoto, T. & Cledón, M. 2012 *Schizymenia dubyi* (Rhodophyta, Schizymeniaceae), a new introduced species in Argentina. New Zeal. J. Bot. 50:51–8.

[jpy12957-bib-0046] Ramírez, M. & Santelices, B. 1991 Catálogo de las algas marinas bentónicas de la costa temperada del Pacífico de Sudamérica. Monografías Biológicas 5, Pontificia Universidad Católica, Santiago, 437 pp.

[jpy12957-bib-0047] Rodriguez, E. C. & Moliner, C. C. 2010 Checklist of benthic algae from the Asturias coast (North of Spain). Bol. Cien. R.I.D.E.A. 51:135–212.

[jpy12957-bib-0048] Ronquist, F. , Teslenko, M. , van der Mark, P. , Ayres, D. , Darling, A. , Höhna, S. , Larget, B. , Liu, L. , Suchard, M. A. & Hulsenbeck, J. P. 2011 MrBayes 3.2: Efficient Bayesian phylogenetic inference and model choice across a large model space. Syst. Biol. 61:539–42. 10.1093/sysbio/sys029 PMC3329765 22357727

[jpy12957-bib-0049] Rueness, J. 1977 Norsk algeflora. Universitetsforlaget, Oslo, 265 pp.

[jpy12957-bib-0050] Saunders, G. W. 2005 Applying DNA barcoding to red macroalgae a preliminary appraisal holds promise for future applications. Philos. Trans. R. Soc. Lond. B Biol. Sci. 360:1879–88. 16214745 10.1098/rstb.2005.1719 PMC1609223

[jpy12957-bib-0051] Saunders, G. W. , Birch, T. C. & Dixon, K. R. 2015 A DNA barcode survey of *Schizymenia* (Nemastomatales, Rhodophyta) in Australia and British Columbia reveals overlooked diversity including *S. tenuis* sp. nov. and *Predaea borealis* sp. nov. Botany 93:859–71.

[jpy12957-bib-0052] Saunders, G. W. & Moore, T. E. 2013 Refinements for the amplification and sequencing of red algal DNA barcode and RedToL phylogenetic markers: a summary of current primers, profiles and strategies. Algae 28:31–43.

[jpy12957-bib-0053] Sciuto, M. , Piattelli, M. , Chillemi, R. , Furnari, G. & Cormaci, M. 1979 The implication of *Haematocelis rubens* J. Agardh in the life history of *Schizymenia dubyi* (Chauvin) J. Agardh (Rhodophyta, Gigartinales): a chemical study. Phycologia 18:296–302.

[jpy12957-bib-0054] Sejrup, H. P. , Hjelstuen, B. O. , Dahlgren, K. I. T. , Haflidason, H. , Kuijpers, A. & Nygård, A. 2005 Pleistocene glacial history of the NW European continental margin. Mar. Pet. Geol. 22:1111–29.

[jpy12957-bib-0055] Setchell, W. A. & Gardner, N. L. 1924 New marine algae from the Gulf of California. Proc. Cal. Acad. Sci. Ser. 4, 12: 695–949, 77 plates.

[jpy12957-bib-0056] Silva, P. C. , Basson, P. & Moe, R. 1996 Catalogue of the benthic marine algae of the Indian Ocean. University of California publications in Botany, Vol 79 University of California Press, Berkeley, CA, 1260 pp.

[jpy12957-bib-0057] Tamura, K. , Peterson, D. , Peterson, N. , Stecher, G. , Nei, M. & Kumar, S. 2011 MEGA5: Molecular evolutionary genetics analysis using maximum likelihood, evolutionary distance, and maximum parsimony methods. Mol. Biol. Evol. 28:2731–9. 21546353 10.1093/molbev/msr121 PMC3203626

[jpy12957-bib-0058] Taylor, W. R. 1945 Pacific marine algae from the Allan Hancock expeditions to the Galapagos Islands. A. Hancock Pacific Exped. 12:1–528.

[jpy12957-bib-0059] Thiers, B. 2018 Index Herbariorum: A global directory of public herbaria and associated staff. New York Botanical Garden's Virtual Herbarium. http://sweetgum.nybg.org/science/ih/, searched on March 22, 2018.

[jpy12957-bib-0060] Vermeij, G. J. 1991 Anatomy of an invasion: the trans‐Arctic interchange. Paleobiology 17:281–307.

