# Supplementary material for: Schizymenia jonssonii sp. nov. (Nemastomatales, Rhodophyta): a relict or an introduction into the North Atlantic after the last glacial maximum?
Source: J Phycol. 2020 Jan 9;56(2):324–33. doi: 10.1111/jpy.12957 (PMC7187443; doi:10.1111/jpy.12957)
Supplement: Supplementary file 1 — Table S1. GenBank accession‐numbers, species, voucher identity, sampling localities, collectors and collecting dates for specimens used in molecular phylogenetic analysis. Sequences that were generated in the present study are in bold. [file JPY-56-324-s001.docx]

Table S1. GenBank accession-numbers, species, voucher identity, sampling localities, collectors and collecting dates for specimens used in molecular phylogenetic analysis. Sequences that were generated in the present study are in bold.

| **Genbank accession no** | |  | **Isolate/** |  |  |
| --- | --- | --- | --- | --- | --- |
| **COI** | ***rbc*L** | **Tentative species name** | **Voucher code** | **Collection site** | **Collector and collecting date** |
| KM254233 |  | S. sp._1Cal | GWS021206 | USA: Montara, CA | B. Clarkston & K.R. Hind, 14-May-2010 |
| KM254422 |  | S. sp._1Cal | GWS021885 | USA: Santa Cruz (Four Mile), CA | B. Clarkston & K.R. Hind, S. Toews, 19-May-2010 |
| KM254362 |  | S. sp._1Cal | GWS022008 | USA: Santa Cruz (Four Mile), CA | B. Clarkston & K.R. Hind, S. Toews, 19-May-2010 |
|  | KP733881 | S. sp_1Cal | GWS021885 | USA: Santa Cruz (Four Mile), CA | B. Clarkston, K.R. Hind & S. Toews, 19-May-2010 |
|  | KP733877 | S. sp_1Cal | GWS022008 | USA: Santa Cruz (Four Mile), CA | B. Clarkston, K.R. Hind & S. Toews, 19-May-2010 |
|  | FJ878871 | S. sp_1Cal (*S. pacifica*) | TC130 | USA: North Boardman St. Park, OR | T.O. Cho & G.I. Gayle, 24-Jul-1998 |
|  |  |  |  |  |  |
| KP725100 |  | *S. tenuis* | GWS010869 | Canada: Bamfield, Blowhole at Brady’s Beach, BC | G.W. Saunders & B. Clarkston, 06-Jun-2008 |
| KP725157 |  | *S. tenuis* | GWS035307 | Canada: Point between Tana and Gudal Bays, Haida Gwaii, BC | G.W. Saunders & K. Dixon, 23-Aug-2013 |
| KP725127 |  | *S. tenuis* | GWS010370 | Canada: Palliser Rock, Comox, BC | B. Clarkston, D.C. McDevit & K.R. Hind, 29-May-2008 |
| KP725119 |  | *S. tenuis* | GWS008351 | Canada: Ridley Island (south of coal terminal), Prince Rupert, BC | G.W. Saunders, B. Clarkston, D.C. McDevit & K. Roy, 08-Jun-2007 |
|  | KP73386 | *S. tenuis* | GWS009944 | Canada: Tahsis, Island #40 on Esperenza Inlet Chart, BC | G. W. Saunders & B. Clarkston, 21-May-2008 |
|  | KP733874 | *S. tenuis* | GWS009969 | Canada: Tahsis, Island #40 on Esperenza Inlet Chart, BC | G.W. Saunders & B. Clarkston, 21-May-2009 |
|  |  |  |  |  |  |
| KP725093 |  | *S. pacifica* | GWS002177 | Canada: Bear Cove Park, Port Hardy, Vancouver Island, BC | G.W. Saunders, C.E. Lane, 17-Jun-2004 |
| KP725112 |  | *S. pacifica* | GWS028182 | Canada: Murchison Is., Northwest Beach, Gwaii Haanas, BC | G.W. Saunders & K. Dixon, 07-Jul-2011 |
| KP725146 |  | *S. pacifica* | GWS003202 | Canada: Land’s End, up the left side of Pachena Bay, Bamfield, BC | G.W. Saunders, R. Withall, 15-Sep-2005 |
|  | AY294394 | *S. pacifica* |  | Canada: Vancouver, Canada, BC | S.C. Lindstrom, 15-Apr-1994 |
|  | JX969800 | *S. pacifica* | GWS012735 | Canada: Burnaby Island near Saw Reef, Gwaii Haanas, BC | G.W. Saunders, D.C. McDevit, 19-Jun-2009 |
|  | AY294393 | *S. pacifica* |  | USA: Kanaha Bay, W. Juan Island, WA | M.J. Wynne, 26-Jul-1995 |
|  |  |  |  |  |  |
| KC478073 |  | *S. apoda* | (mbccc52) | China: Shandong 266003, Qingdao | X. Zhao submitted 10-Jan-2013 |
| KP725129 |  | *S. apoda* | GWS029386 | Australia: Warrnambool Boat ramp, Victoria | G.W. Saunders, 12-Nov-2011 |
| KP725101 |  | *S. apoda* | GWS029381 | Australia: Warrnambool Boat ramp, Victoria | G.W. Saunders, 21-May-2010 |
|  | AY294401 | *S. apoda (S. obovata)* |  | Namibia: Swakopmund | M.H. Hommersand, 06-Jul-1993 |
|  | FJ878865 | *S. apoda* | isolate SMG-05-145 | Portugal: Sao Roque, Sao Miguel, Azores | D. Gabriel, 09-Aug-2005 |
|  | FJ878861 | *S. apoda* | isolate GRW-04-88 | Portugal: Barro Vermelho, Graciosa, Azores | D. Gabriel, 10-Jun-2004 |
|  | KP733876 | *S. apoda* | GWS029381 | Australia: Warrnambool Boat ramp, Victoria | G.W. Saunders, 12-Nov-2011 |
|  | KP733885 | *S. apoda*, sporophyte | GWS029386 | Australia: Warrnambool Boat ramp, Victoria | G.W. Saunders, 12-Nov-2012 |
|  | KP733882 | *S. apoda* | GWS033914 | Australia: Whale Cove, Currarong, Jervis Bay, New South Wales | K. Dixon, 05-Dec-2012 |
|  | KP733870 | *S. apoda*, sporophyte | GWS029477 | Australia: breakwater at harbour, Portland, Victoria | G.W. Saunders & K. Dixon, 14-Nov-2011 |
|  | AY294392 | *S. apoda* |  | China: Taiping Cape, Shandong Province | M.H. Hommersand, submitted 09-May-2003 |
|  | **MN567256** | ***S. apoda*** |  | **MBA shore, Plymouth, UK, 50.364°N, 4.141°W** | **Juliet Brodie, 13-Mar-2013** |
|  |  |  |  |  |  |
| KM254443 |  | *S. dubyi* | GWS022214 | USA: McAbee Beach, Monterey, CA | G.W. Saunders, 12-Nov-2013 |
| KP725131 |  | *S. dubyi* | GWS014213 | Australia: Williamstown, Victoria | J. A. Lewis, 28-Jan-2011 |
| KP725141 |  | *S. dubyi* | GWS032460 | Australia: Manly Beach (rocks at north end), New South Wales | G.W. Saunders & K. Dixon, 24-Nov-2012 |
| KP725138 |  | *S. dubyi* | GWS027573 | Italy: Maria La Scala, Acireale, Sicily | J. Utge & L. LeGall, 24-Nov-2012 |
|  | KP733865 | *S. dubyi* | GWS027574 | Italy: Maria La Scala, Acireale, Sicily | J. Utge & L. Le Gall, 10-May-2011 |
|  | FJ013041 | *S. dubyi* |  | USA: Monterey, California | J.R. Hughey, 13-Sep-2006 |
|  | AB564325 | *S. dubyi* |  | Chile: Navidad, La Boca | M.E. Ramirez, 12-Jan-2002 |
|  | AB564326 | *S. dubyi* |  | Japan: Niigata, Sado, Mano, Takiwaki | M. Suzuki, 28-Feb-2003 |
|  | AY294389 | *S. dubyi* |  | France: Piguet, Brittany | J. Cabioch, submitted 09-May-2003 |
|  | AB564324 | *S. dubyi* |  | Argentina: Mar del Plata | M.E. Ramirez et al., 21-Sep-2008 |
|  | KP733880 | *S. dubyi* | GWS002456 | Australia: Boat ramp behind breakwater, Warrnambool, Victoria | G.W. Saunders & G.T. Kraft, 17-Oct-2004 |
|  |  |  |  |  |  |
|  | AY294391 | S. sp? |  | Japan, | S. Fredericq, submitted 09-May-2003 |
|  | AY294390 | S. sp? |  | Japan: Oshoro, | S. Fredericq, 05-Sep-1995 |
|  |  |  |  |  |  |
| **MN567249** | **MN567255** | ***S. jonssonii*** | **ICEL5343** | **Iceland: Hrísey, 66.028°N, 18.408°W** | **K. Gunnarsson & J. Brodie, 14-Jun-2006** |
| **MN567250** | **MN567257** | ***S. jonssonii*** | **ICEL7748** | **Iceland: Skalavik, 66.186°N, 23.479°W** | **K. Gunnarsson & S. Egilsdottir, 03-Jun-2008** |
| **MN567251** | **MN567258** | ***S. jonssonii*** |  | **Iceland: Flatey, 65.376°N, 22.919°W** | **K. Gunnarsson, 02-Jul-2006** |
| **MN567252** | **MN567259** | ***S. jonssonii*** | **BM013844101** | **Iceland: Stekkjarvikur, 64.029°N, 22.239°W** | **K. Gunnarsson, 21-Apr-2016** |
| **MN567253** | **MN567260** | ***S. jonssonii,* sporophyte** | **BM013844102** | **Iceland: Stekkjarvikur, 64.029°N, 22.239°W** | **K. Gunnarsson, 21-Apr-2016** |
| **MN567254** |  | ***S. jonssonii*** | **ICEL12703** | **Iceland: Kalmanstjarnarvik, 63.897°N, 22.711°W** | **K. Gunnarsson & S. Egilsdottir, 04-Jul-2015** |
|  |  |  |  |  |  |
| JN659917 |  | *Platoma cyclocolpum* | GWS000133 | Spain: Gran Canaria, Canary Islands | M.D. Guiry, submitted 08-Sep-2011 |
|  | AY294385 | *Platoma cyclocolpum* |  | Spain: Canary Islands | Y. DeJong, submitted 09-May-2003 |
|  |  |  |  |  |  |
| HM915966 |  | *Titanophora weberae* | GWS002018 | Australia: Tasmanian Sea, Lord Howe Island, New S-Wales | G.T. Kraft, 30-Jan-2004 |
|  | KP733887 | *Titanophora weberae* | GWS002018 | Australia: North Head Gutters, Lord Howe Island, New S-Wales | G.T. Kraft, 30-Jan-2004 |
